# Zooming in: From spatially extended traveling waves to localized structures: The case of the Sine-Gordon equation in (1+3) dimensions

**DOI:** 10.1371/journal.pone.0175783

**Published:** 2017-04-19

**Authors:** Yair Zarmi

**Affiliations:** Jacob Blaustein Institutes for Desert Research, Ben-Gurion University of the Negev, Midreshet Ben-Gurion, Israel; São Paulo State University, BRAZIL

## Abstract

The Sine-Gordon equation in (1+3) dimensions has *N*-traveling front (“kink”, “domain wall”)- solutions for all *N* ≥ 1. A nonlinear functional of the solution, which vanishes on a single-front, maps multi-front solutions onto sets of infinitely long, but laterally bounded, rods, which move in space. Each rod is localized in the vicinity of the intersection of two Sine-Gordon fronts. The rod systems are solutions of the linear wave equation, driven by a term that is constructed out of Sine-Gordon fronts. An additional linear operation maps multi-rod systems onto sets of blobs. Each blob is localized in the vicinity of rod intersection, and moves in space. The blob systems are solutions of the linear wave equation, driven by a term that is also constructed out of Sine-Gordon fronts. The temporal evolution of multi-blob solutions mimics elastic collisions of systems of spatially extended particles.

## 1. Introduction

### 1.1 Review of paper

A large body of literature is focused on the search for non-linear evolution equations in (1+*n*) dimensions, *n* ≥1, the solutions of which are localized in space and time. This note presents a systematic approach to the generation of dynamical systems that have localized solutions out of known equations, the solutions of which are spatially extended in (1+*n*) dimensions. The example chosen is that of the Sine-Gordon equation in (1+3) dimensions:

$$\partial_{\mu }\partial^{\mu }u\;+\text{ sin}u=\partial_{t}{}^{2}u\;-\;\vec{\nabla }\cdot \vec{\nabla }u\;+\text{ sin}u=0.$$ (1)

(In the notation of Eq (1) the speed of light is *c* = 1.)

**Fig 1:**
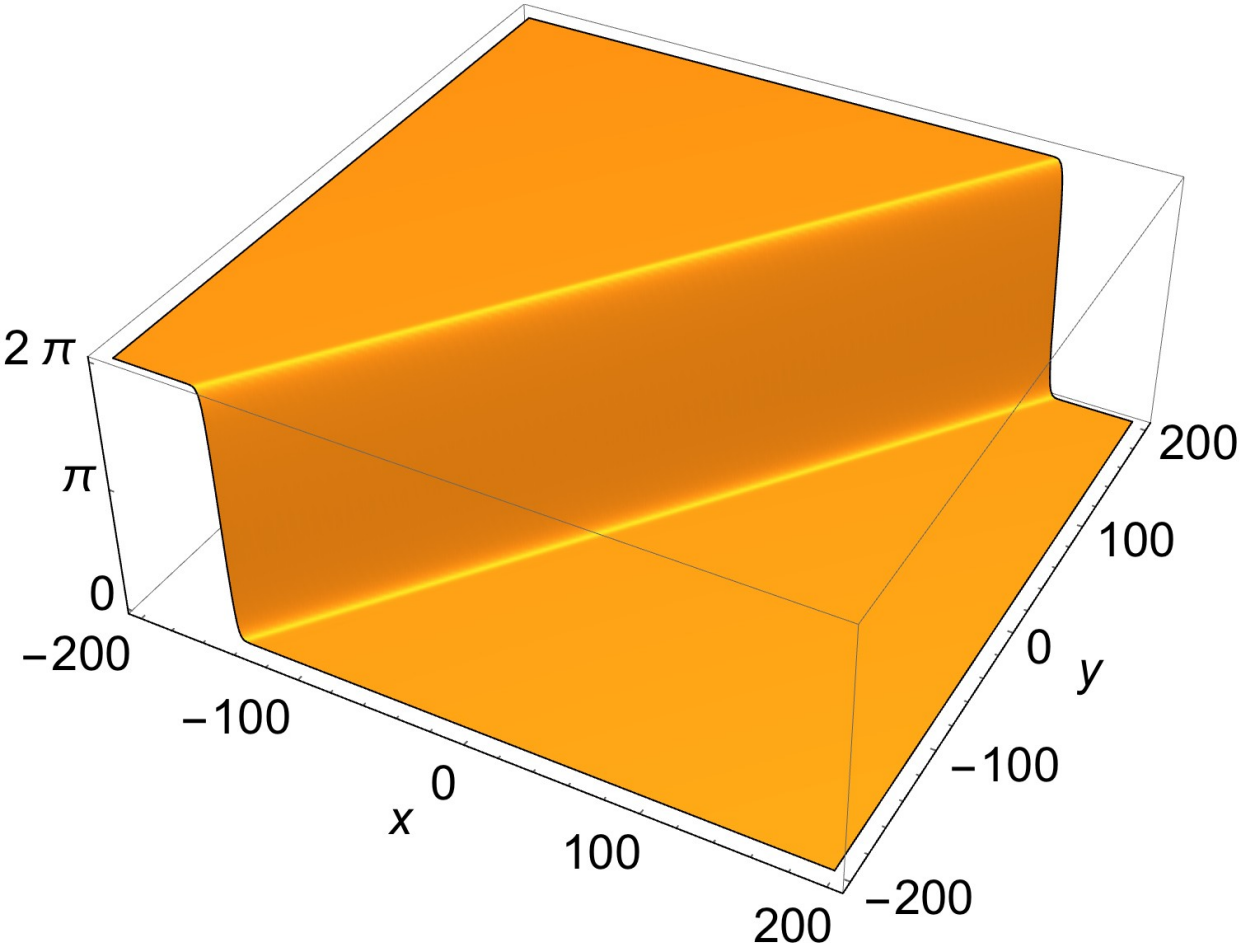
Single-front solution of Eq (1) in rest frame. *p* = {0, cos*φ*, sin*φ*, 0}; *φ* = −*π*/5; *δ* = 0.

**Fig 2:**
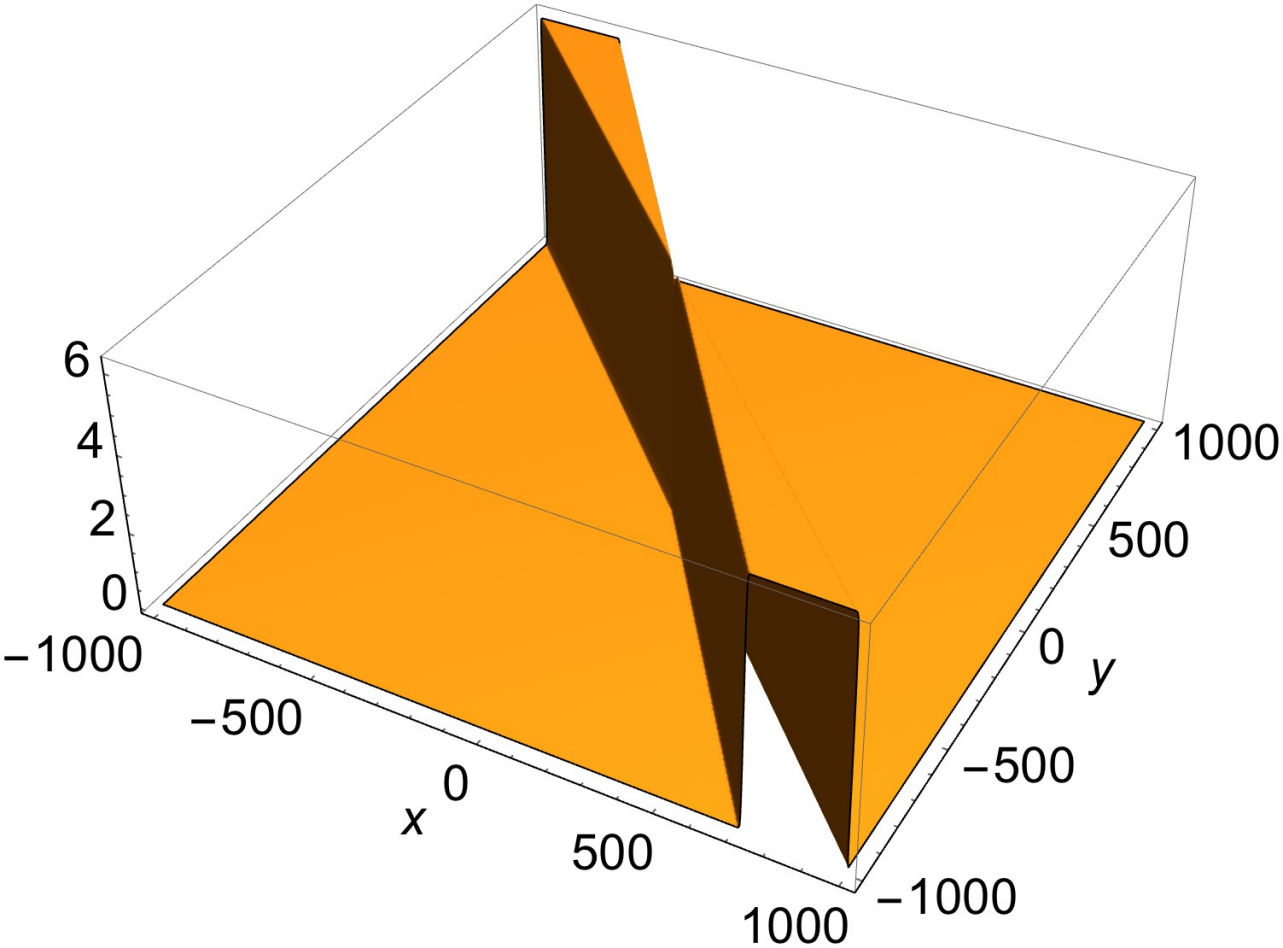
Two-front solution of Eq (1) at *t* = *z* = 0. *p*^(1)^ = {1, cos*φ*^(1)^, sin*φ*^(1)^, 1}, *p*^(2)^ = {1, cos*φ*^(2)^, sin*φ*^2^, 1}; *φ*^(1)^ = *π*/5, *φ*^(2)^ = *π*/4; *δ*^(1)^ = *δ*^(2)^ = 0.

**Fig 3:**
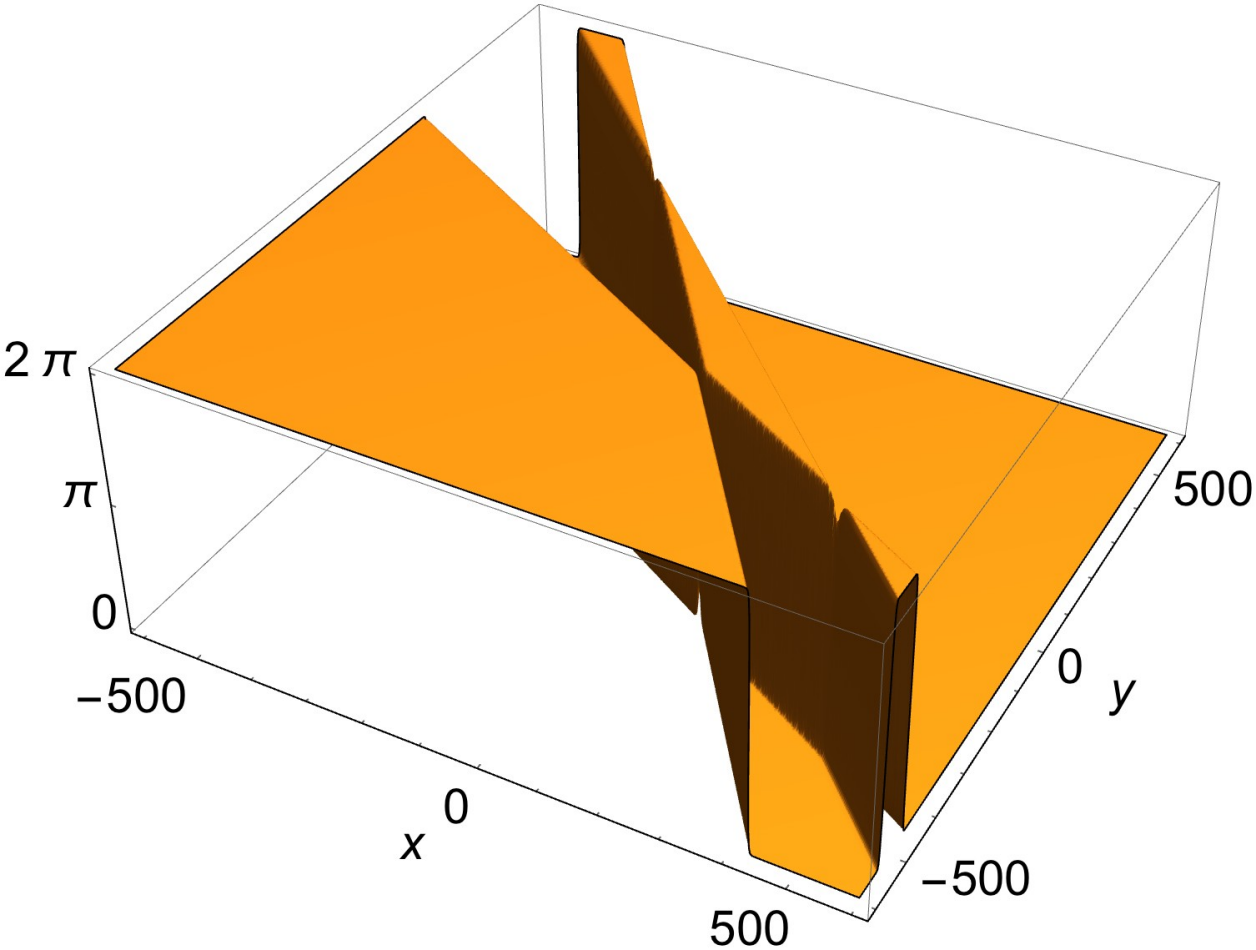
Three-front solution of Eq (1) at *t* = 0, *z* = 50. *p*^(1)^ = {0, cos*φ*^(1)^, sin*φ*^(1)^, 0}, *p*^(2)^ = {0, cos*φ*^(2)^, sin*φ*^2^, 0}, *p*^(3)^ = {1, cos*φ*^(3)^, sin*φ*^(3)^, 1}, *φ*^(1)^ = *π*/5, *φ*^(2)^ = *π*/4, *φ*^(3)^ = *π*/3; *δ*^(1)^ = 0, *δ*^(2)^ = 50, *δ*^(3)^ = −50.

Despite the fact that in more than one space dimension the Sine-Gordon equation is not integrable [1–5], Eq (1) has *N*-front solutions for all *N* ≥ 1 (also called “kinks” or “domain walls”). Their construction through the Hirota algorithm [6–8] as well the wealth of solutions and their properties (studied in Ref [9]) are reviewed in Section 2. An important property of multi-front solutions is that in the vicinity of their intersections with one another fronts lose their identity, but away from the intersection regions, each front tends asymptotically to a single-front solution. Examples of solutions with one, two and three fronts are shown in, Figs 1, 2 and 3, respectively.

Consider now the following nonlinear functional of the solution of Eq (1):

$$R\left[u\right]=\frac{1}{2}\;\partial_{\mu }u\;\partial^{\mu }u\;+\;\left(1-\text{ cos}u\right).$$ (2)

*R*[*u*], has been defined and studied in the case of the (1+2)-dimensional version of Eq (1), and shown to vanish identically when *u* is a single-front solution [10]. Repetition of the arguments shows that it vanishes identically when *u* is a single-front solution in any space dimension. When *u* represents *N* ≥ 2 fronts, *R*[*u*] is non-zero in the neighborhood of front intersections. Away from intersection regions, it vanishes exponentially fast along each front. As a result, in (1+3) dimensions, *R*[*u*] is comprised of infinitely long, laterally bounded rods that are localized in the vicinity of front intersections.

Repeated application of Eq (1) yields that *R*[*u*] obeys the linear wave equation, driven by a term that is constructed from the solution of Eq (1):

$$\partial_{\mu }\partial^{\mu }R=\partial_{\mu }\partial_{\nu }u\;\partial^{\mu }\partial^{\nu }u\;-\;{\left(\partial_{\mu }\partial^{\mu }u\right)}^{2}.$$ (3)

The properties of rod solutions of Eq (3) are discussed in detail in Section 3. The following is a review of some outstanding properties.

**Fig 4:**
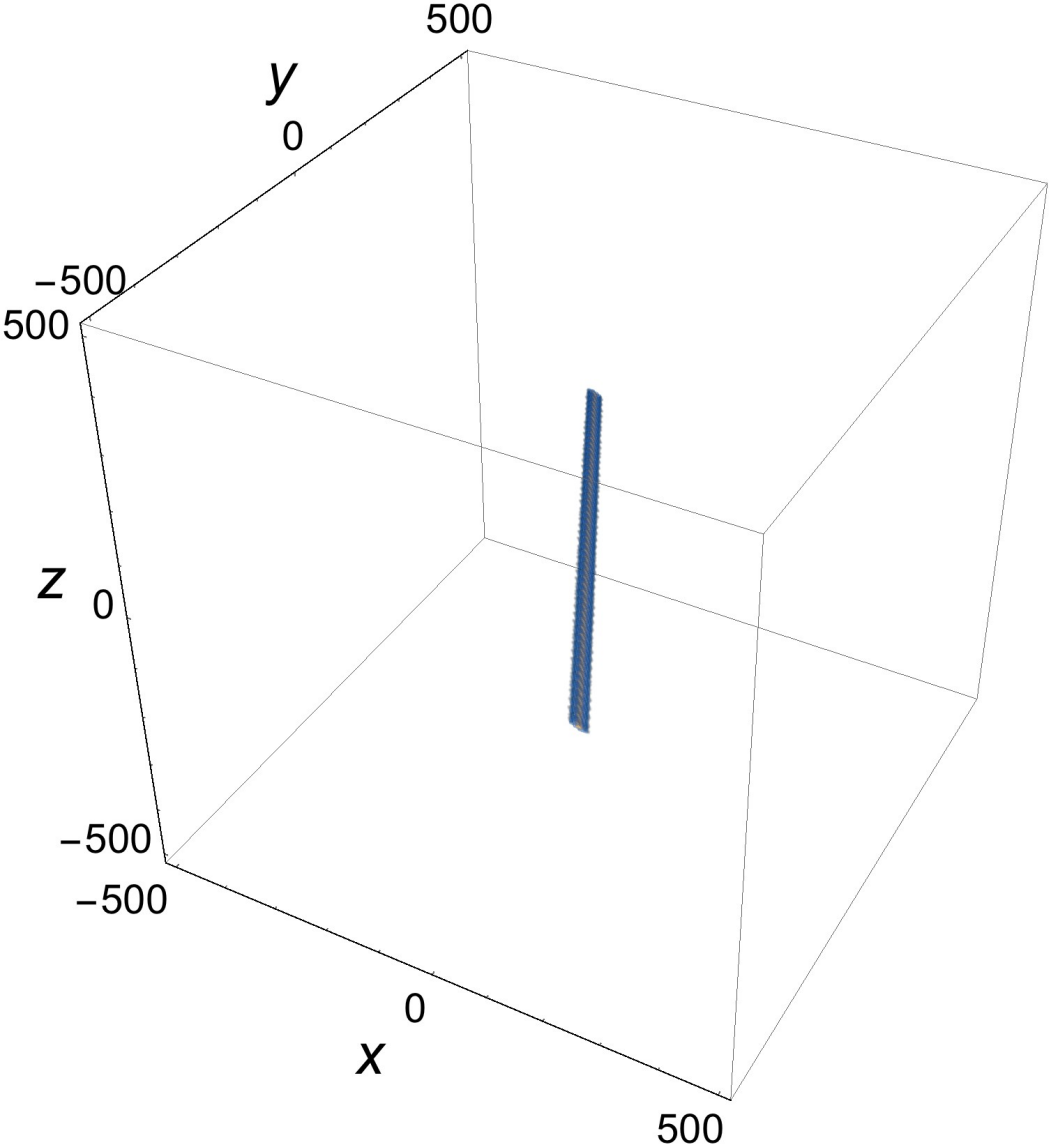
Single-rod solution of Eq (3) at *t* = 0. *p*^(1)^ = {1, cos*φ*^(1)^, sin*φ*^(1)^, 1}, *p*^(2)^ = {2, cos*φ*^(2)^, sin*φ*^(2)^, 2}; *φ*^(1)^ = *π*/5, *φ*^(2)^ = *π*/4; *δ*^(1)^ = *δ*^(2)^ = 0.

**Fig 5:**
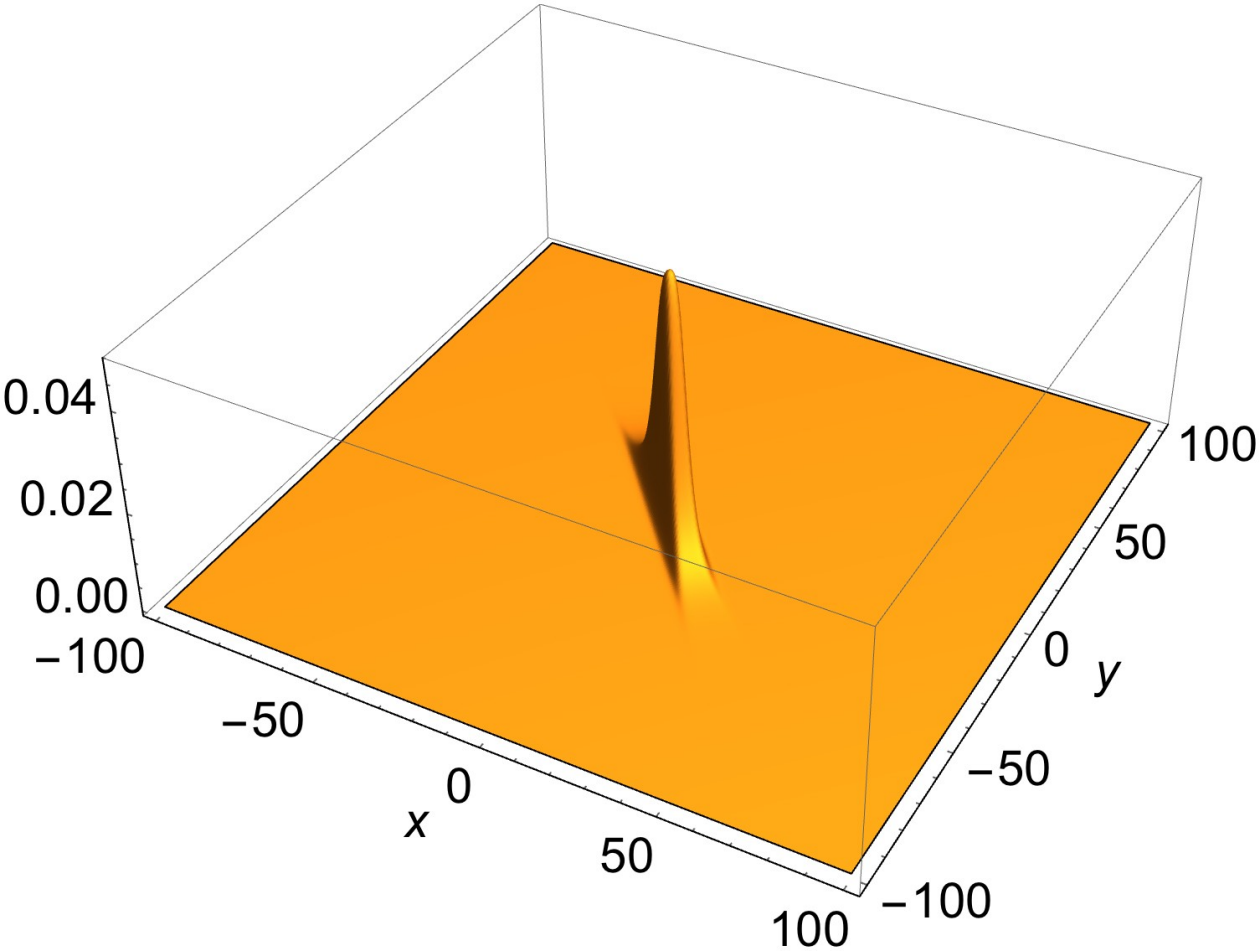
Lateral profile of single-rod solution of Eq (3) in rest frame. *p*^(1)^ = {0, cos*φ*^(1)^, sin*φ*^(1)^, 0}, *p*^(2)^ = {0, cos*φ*^(2)^, sin*φ*^(2)^, 0}; *φ*^(1)^ = *π*/5, *φ*^(2)^ = *π*/4; *δ*^(1)^ = *δ*^(2)^ = 0.

A single-rod solution of Eq (3) is generated when *u* is a two-front solution of Eq (1). An example of a single-rod solution is shown in Fig 4. The profile of *R*[*u*] does not depend on the distance along its longitudinal axis. It is a function only of the lateral coordinates, which falls off exponentially fast away from the longitudinal axis. An example of the dependence of the profile on the lateral coordinates is presented in Fig 5. Finally, the rod propagates at a constant speed in a direction perpendicular to its longitudinal axis.

**Fig 6:**
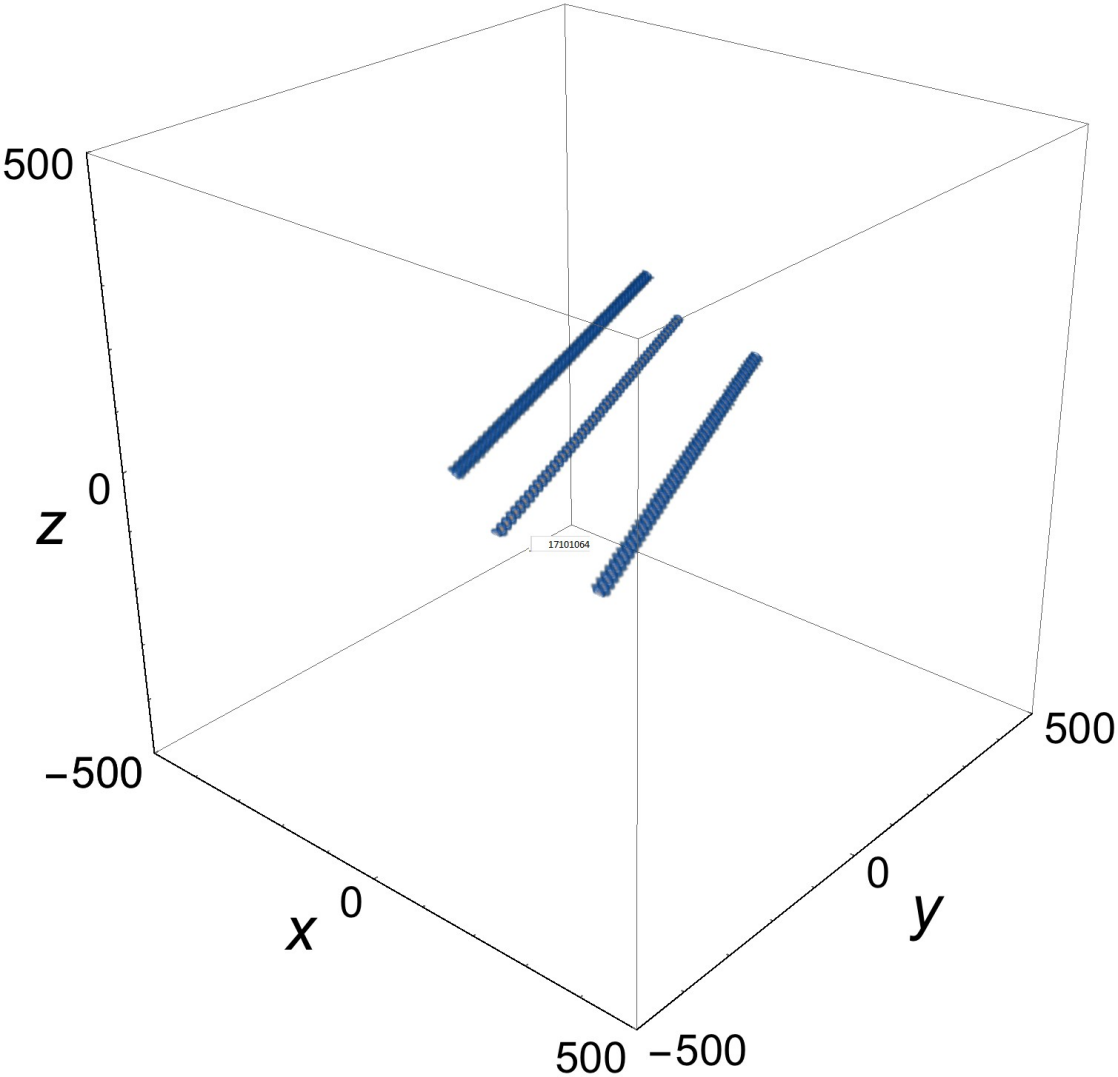
Three parallel-rod solution of Eq (3) at *t* = 0. *p*^(1)^ = {1, cos*φ*^(1)^, sin*φ*^(1)^, 1}, *p*^(2)^ = {2, cos*φ*^(2)^, sin*φ*^(2)^, 2}; *p*^(3)^ = {*p*0, cos*φ*^(3)^, sin*φ*^(3)^, *p*0} = *αp*^(1)^ + *βp*^(2)^; *α* = −1.65449; *β* = 2.60004; *φ*^(1)^ = *π*/5, *φ*^(2)^ = *π*/4, *φ*^(3)^ = *π*/3*δ*^(1)^ = 0, *δ*^(2)^ = 50, *δ*^(3)^ = −50.

**Fig 7:**
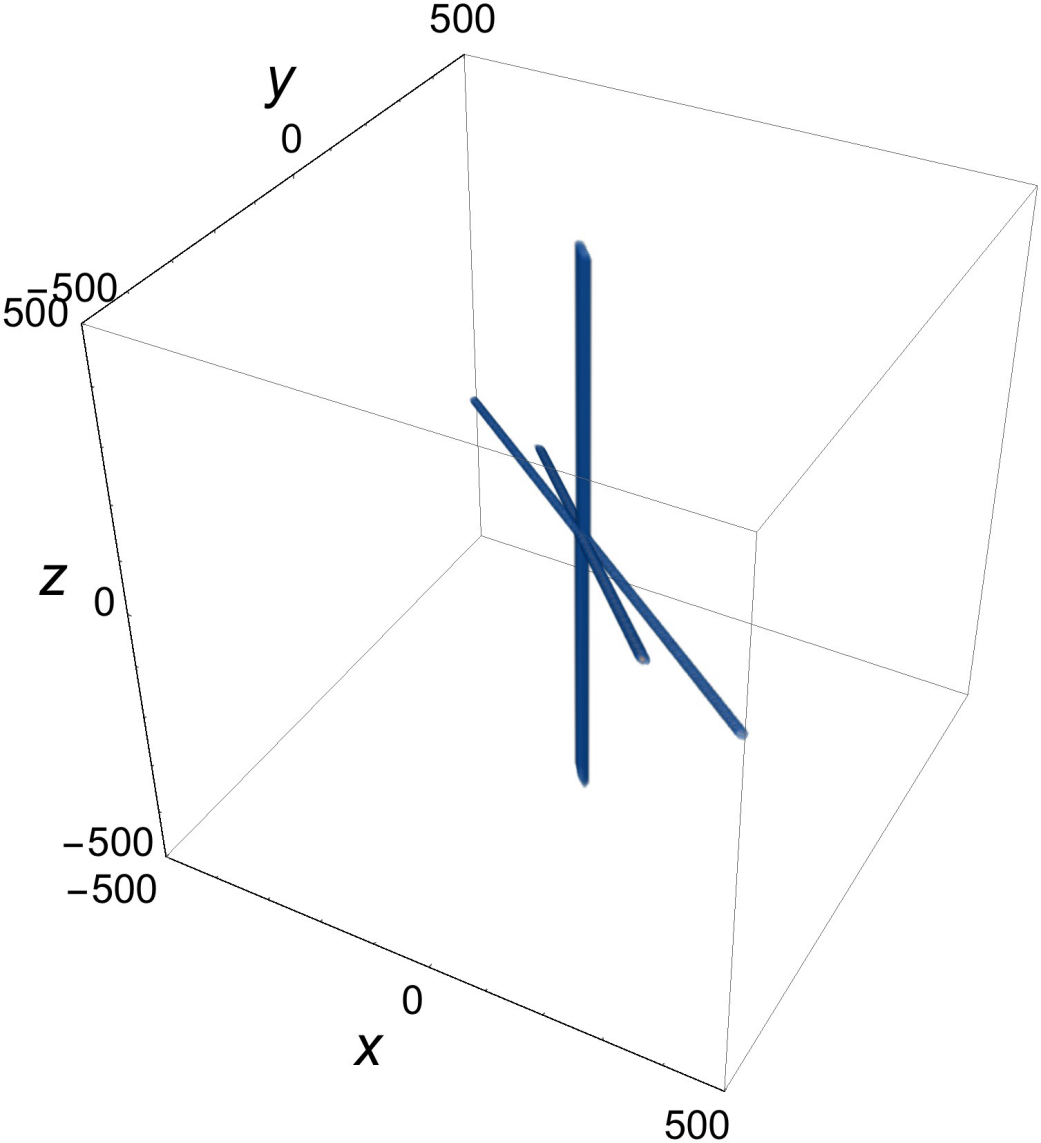
Three intersecting rod solution of Eq (3). *p*^(1)^ = {0, cos*φ*^(1)^, sin*φ*^(1)^, 0}, *p*^(2)^ = {0, cos*φ*^(2)^, sin*φ*^2^, 0}, *p*^(3)^ = {1, cos*φ*^(3)^, sin*φ*^(3)^,1}; *φ*^(1)^ = *π*/5, *φ*^(2)^ = *π*/4, *φ*^(3)^ = *π*/3; *δ*^(1)^ = *δ*^(2)^ = *δ*^(3)^ = 0.

In a multi-rod solution of Eq (3), the rods may be parallel or intersect. If they intersect, they lose their identity around the intersection regions. Away from the intersections, each rod tends to a single-rod solution. Examples of three-rod solutions with parallel and with intersecting rods are shown, respectively, in Figs 6 and 7.

*R*[*u*] maps the spatially extended multi-front solutions of Eq (1) onto rods, which are confined to the vicinity of front intersections. A similar idea is developed in Section 4 for the case of the rods. A linear operation maps multi-rod solutions of Eq (3) onto sets of blobs that are localized around rod intersections. The system of blobs is also a solution of the linear wave equation, driven by a term that is constructed from the solution of Eq (1). It mimics a system of spatially extended particles that undergo elastic collisions.

### 1.2 Additional motivation: Coupled Klein-Gordon equations

There is very broad interest in systems of coupled nonlinear Klein-Gordon equations [11–21], and recently, in the study of the dynamics of deformed DNA chains [22, 23]. All these studies have been confined to (1+1) and (1+2) dimensions. The system of Eqs (1) and (3) provides a (1+3) dimensional example, which emerges naturally from properties of the solutions of Eq (1).

## 2. Front solutions of Sine-Gordon equation in (1+3) dimensions

### 2.1 Construction of solutions

An *N*-front solution of Eq (1)) is constructed in terms of two auxiliary functions [6,7]:

$$u\left(x;\;P\right)=4\;{\text{tan}}^{-1}\left[g\left(x;\;P\right)/f\left(x;\;P\right)\right],$$ (4)

$$P\equiv \left\{p^{\left(1\right)},\;p^{\left(2\right)},\;\ldots ,\;p^{\left(N\right)}\right\}.$$ (5)

In Eq (4), *x* is the coordinate 4-vetor, and *p*^(*i*)^, *i* = 1, 2,..*N*, are momentum 4-vector parameters. The functions *g*(*x*;*P*) and *f*(*x*;*P*) are given by:

$$g\left(x;\;P\right)=\underset{\begin{array}{c} 1\leq n\leq N\\ n\;odd \end{array}}{\sum }\left(\underset{1\leq i_{1}<\cdots <i_{n}\leq N}{\sum }\left\{\overset{n}{\underset{j=1}{\prod }}\varphi \left(x;\;p^{\left(i_{j}\right)}\right)\;\underset{i_{l}<i_{m}}{\prod }V\left(p^{\left(i_{l}\right)},\;p^{\left(i_{m}\right)}\right)\right\}\right),$$ (6)

$$f\left(x;\;P\right)=1\;+\;\underset{\begin{array}{c} 2\leq n\leq N\\ n\;even \end{array}}{\sum }\left(\underset{1\leq i_{1}<\cdots <i_{n}\leq N}{\sum }\left\{\overset{n}{\underset{j=1}{\prod }}\varphi \left(x;\;p^{\left(i_{j}\right)}\right)\;\underset{i_{l}<i_{m}}{\prod }V\left(p^{\left(i_{l}\right)},\;p^{\left(i_{m}\right)}\right)\right\}\right),$$ (7)

where

$$\varphi \left(x;\;p^{\left(i\right)}\right)=e^{\xi_{i}\;+\;\delta_{i}},\;\left(\xi_{i}=p^{\left(i\right)}{}_{\mu }\;x^{\mu }\right),$$ (8)

$$p^{\left(i\right)}{}_{\mu }\;p^{\left(i\right)}{}^{\mu }=-1.$$ (9)

In Eq (8), *δ*_*i*_ is a constant arbitrary phase. Finally,

$$V\left(p,\;p\prime \right)=\frac{{\left(p\;-\;p\prime \right)}_{\mu }\;{\left(p\;-\;p\prime \right)}^{\mu }}{{\left(p\;+\;p\prime \right)}_{\mu }\;{\left(p\;+\;p\prime \right)}^{\mu }}=\frac{1\;+\;p_{\mu }\;p\prime^{\mu }}{1\;-\;p_{\mu }\;p\prime^{\mu }}.$$ (10)

The lack of integrability of the Sine-Gordon equation beyond (1+1) dimensions [1–5] shows up through a constraint on the parameter vectors, *p*^(*i*)^, in *N*-front solutions for all *N* ≥ 3 [8]. The components of each triplet of vectors (total of $\left(\begin{array}{l} N\\ 3 \end{array}\right)$ triplets) must obey the constraint:

$${\left(\Delta_{0}\right)}^{2}={\left(\Delta_{x}\right)}^{2}\;+\;{\left(\Delta_{y}\right)}^{2}\;+\;{\left(\Delta_{z}\right)}^{2}.$$ (11)

Denoting by *p*^(*i*)^, *p*^(*j*)^, and *p*^(*k*)^ (1 ≤ *i* ≠ *j* ≠ *k* ≤ *N*) the three vectors in a triplet, *Δ*_0_, *Δ*_*x*_, *Δ*_*y*_ and *Δ*_*z*_ are defined by:

$$\begin{array}{ccc} \Delta_{x}=\left|\begin{array}{ccc} p_{0}^{\left(i\right)} & p_{y}^{\left(i\right)} & p_{z}^{\left(i\right)}\\ p_{0}^{\left(j\right)} & p_{y}^{\left(j\right)} & p_{z}^{\left(j\right)}\\ p_{0}^{\left(k\right)} & p_{y}^{\left(k\right)} & p_{z}^{\left(k\right)} \end{array}\right|, & \Delta_{y}=\left|\begin{array}{ccc} p_{0}^{\left(i\right)} & p_{z}^{\left(i\right)} & p_{x}^{\left(i\right)}\\ p_{0}^{\left(j\right)} & p_{z}^{\left(j\right)} & p_{x}^{\left(j\right)}\\ p_{0}^{\left(k\right)} & p_{z}^{\left(k\right)} & p_{x}^{\left(k\right)} \end{array}\right|, & \Delta_{z}=\left|\begin{array}{ccc} p_{0}^{\left(i\right)} & p_{x}^{\left(i\right)} & p_{y}^{\left(i\right)}\\ p_{0}^{\left(j\right)} & p_{x}^{\left(j\right)} & p_{y}^{\left(j\right)}\\ p_{0}^{\left(k\right)} & p_{x}^{\left(k\right)} & p_{y}^{\left(k\right)} \end{array}\right|\\ & \Delta_{0}=\left|\begin{array}{ccc} p_{x}^{\left(i\right)} & p_{y}^{\left(i\right)} & p_{z}^{\left(i\right)}\\ p_{x}^{\left(j\right)} & p_{y}^{\left(j\right)} & p_{z}^{\left(j\right)}\\ p_{x}^{\left(k\right)} & p_{y}^{\left(k\right)} & p_{z}^{\left(k\right)} \end{array}\right| & \end{array},$$ (12)

### 2.2 Review of properties of front solutions [9]

The following discussion hinges on the properties under Lorentz transformations of the momentum vectors employed in the construction of solutions of Eq (1) through Eqs (4)–(10). These properties, summarized in Appendix A, depend crucially on Eq (9).

Each front, be it a single-front solution, or a front in a multi-front solution, once away from front intersections, propagates at a velocity, *v*, that is lower than *c* = 1:

$$v=\left|p_{0}/\left|\vec{p}\right|\right|<c=1.$$ (13)

A two-front solution depends on *x*, the coordinate 4-vector, through two Lorentz scalars, *ξ*_1_ and *ξ*_2_ (see Eq (8)). As a result, the following vector operation on a two-front solution vanishes:

$$J^{\mu }\left[u\right]=\varepsilon^{\mu \alpha \beta \gamma }\;p_{\alpha }^{\left(1\right)}\;p_{\beta }^{\left(2\right)}\;\partial_{\gamma }u=\varepsilon^{\mu \alpha \beta \gamma }\;p_{\alpha }^{\left(1\right)}\;p_{\beta }^{\left(2\right)}\;\left(p_{\gamma }^{\left(1\right)}\;\partial_{\xi_{1}}u\;+\;p_{\gamma }^{\left(2\right)}\;\partial_{\xi_{2}}u\right)=0,$$ (14)

where *ε*^*μαβγ*^ is the antisymmetric Levi-Civita tensor. In particular, one has

$$J^{0}\left[u\right]={\vec{p}}^{\left(1\right)}\times {\vec{p}}^{\left(2\right)}\cdot \vec{\nabla }u=\left|{\vec{p}}^{\left(1\right)}\right|\;\left|{\vec{p}}^{\left(2\right)}\right|\frac{\partial u}{\partial l}=0.$$ (15)

In Eq (15), *l* is a coordinate along the normal to the plane defined by the space parts, ${\vec{p}}^{\left(1\right)}$ and ${\vec{p}}^{\left(2\right)}$. Thus, apart from its time dependence, the profile of the two-front configuration depends only on the two space coordinates in that plane. It is a (1+2)-dimensional structure.

Finally, a two-front solution propagates rigidly in the plane defined by the ${\vec{p}}^{\left(1\right)}$ and ${\vec{p}}^{\left(2\right)}$, at a constant velocity, *v*. The value of *v* and the range of values of the solution *u* are affected by |*p*^(1)^ · *p*^(2)^|, the magnitude of the scalar product in Minkowski space of the two 4-momenta. One finds:

$$\left|p^{\left(1\right)}\cdot p^{\left(2\right)}\right|<1\Rightarrow v<c,\;V\left(p^{\left(1\right)},\;p^{\left(2\right)}\right)>0,$$ (16)

and

$$\left|p^{\left(1\right)}\cdot p^{\left(2\right)}\right|>1\Rightarrow v>c,\;V\left(p^{\left(1\right)},\;p^{\left(2\right)}\right)<0.$$ (17)

When Eq (16) holds, thanks to the positivity of *V*(*p*^(1)^, *p*^(2)^), each front varies in the range [0, 2*π*]. When Eq (17) holds, one front varies in the range [0, 2*π*], whereas the other front varies in the range [2*π*, 4*π*]. (Equality in Eqs (16) and (17), *p*^(1)^ · *p*^(2)^ = ±1, is of no interest: An *N*-front solution then degenerates to one with {*N*–(3±1)/2} fronts.)

In the case of solutions with *N* ≥ 3 fronts, Eq (11) affects the properties of each triplet of fronts generated by momentum vectors, *p*^(*i*)^, *p*^(*j*)^, and *p*^(*k*)^. These properties depend on whether Δ_0_ of Eq (12) obeys

$$\Delta_{0}=0,$$ (18)

or

$$\Delta_{0}\neq 0.$$ (19)

When Eq (18) holds, Eq (11) implies that one must also have

$$\Delta_{x}=\Delta_{y}=\Delta_{z}=0.$$ (20)

In this case, the three 4-vectors are linearly dependent:

$$p^{\left(k\right)}=\alpha \;p^{\left(i\right)}\;+\;\beta \;p^{\left(j\right)}.$$ (21)

Such a three-front configuration is (1+2)-dimensional. Consider, for example, the case of *N* = 3, with momentum vectors *p*^(1)^, *p*^(2)^, and *p*^(3)^. Thanks, to Eq (21), the Lorentz scalar *ξ*_3_ is now a linear combination of *ξ*_1_ and *ξ*_2_ (see Eq (8)); the solution depends only on these two Lorentz scalars. As a result, Eq (15) holds also for this three-front solution: The profile of the front-triplet is independent of the distance along the line perpendicular to the plane defined by ${\vec{p}}^{\left(1\right)}$ and ${\vec{p}}^{\left(2\right)}$, and the three fronts propagate rigidly in the plane. Eqs (16) or (17) determine the velocity of propagation.

The case of Eq (19) represents another physical situation. In that case, let us define

$$\beta_{x}=\frac{\Delta_{x}}{\Delta_{0}},\;\beta_{y}=\frac{\Delta_{y}}{\Delta_{0}},\;\beta_{z}=\frac{\Delta_{z}}{\Delta_{0}}.$$ (22)

As long as

$$\beta_{x}{}^{2}\;+\;\beta_{y}{}^{2}\;+\;\beta_{z}{}^{2}\leq 1,$$ (23)

*β*_*x*_, *β*_*y*_ and *β*_*z*_ are the components of the velocity of a Lorentz boost, *L*, which transforms the three momenta into pure space-like ones (see Appendix A):

$$p^{\left(i\right)}\underset{L}{\to }\left(0,\;{\vec{q}}^{\left(i\right)}\right)\;{\vec{q}}^{\left(i\right)}\cdot {\vec{q}}^{\left(i\right)}=1\;\left(i=1,\;2,\;3\right).$$ (24)

In the resulting frame of reference, all three fronts are stationary (time-independent), as the time components of the transformed 4-momentum vectors vanish. Eq (11) can be now written as

$$\beta_{x}{}^{2}\;+\;\beta_{y}{}^{2}\;+\;\beta_{z}{}^{2}=1.$$ (25)

Eq (25) means that the velocity of the Lorentz boost is *v* = *c* = 1. Namely, when Eqs (11) and (19) hold, the triplet of fronts propagates rigidly at the speed of light.

## 3. Rod solutions of Eq (3)

### 3.1 Single-rod solution

When *u* is a two-front solution of Eq (1), *R*[*u*], the solution of Eq (3), maps it onto a single rod. The two-front solution depends on two 4-vectors, *p*^(1)^ and *p*^(2)^, through the two Lorentz scalars, *ξ*_1_ and *ξ*_2_ (see Eqs (4)–(10)). As a result, *R*[*u*] is also a function only of *ξ*_1_ and *ξ*_2_ [10]:

$$R\left[u\right]=\frac{32\;e^{\xi_{1}\;+\;\xi_{2}}\;V\left(p^{\left(1\right)},\;p^{\left(2\right)}\right)}{\left(1\;+\;V\left(p^{\left(1\right)},\;p^{\left(2\right)}\right)\right)\;\left\{1\;+\text{​}e^{2\;\xi_{1}}\;+\;e^{2\;\xi_{2}}\;+\;e^{2\;\left(\xi_{1}\;+\;\xi_{2}\right)}\;{\left(V\left(p^{\left(1\right)},\;p^{\left(2\right)}\right)\right)}^{2}\;+\;2\;e^{\xi_{1}\;+\;\xi_{2}}\;\left(1\;+\;V\left(p^{\left(1\right)},\;p^{\left(2\right)}\right)\right)\right\}}.$$ (26)

(Without loss of generality, the constant phase shifts of Eq (8) have been omitted.)

*R*[*u*] is confined to the intersection region of the two fronts and vanishes asymptotically over each front once away from the intersection region. Furthermore, as a consequence of Eq (15), it is independent of the coordinate along its longitudinal axis:

$$\frac{\partial R\left[u\right]}{\partial l}\equiv \frac{\left({\vec{p}}^{\left(1\right)}\times {\vec{p}}^{\left(2\right)}\right)}{\left|{\vec{p}}^{\left(1\right)}\right|\;\left|{\vec{p}}^{\left(2\right)}\right|}\cdot \vec{\nabla }R\left[u\right]=\frac{\left({\vec{p}}^{\left(1\right)}\times {\vec{p}}^{\left(2\right)}\right)}{\left|{\vec{p}}^{\left(1\right)}\right|\;\left|{\vec{p}}^{\left(2\right)}\right|}\cdot \left({\vec{p}}^{\left(1\right)}\;\partial_{\xi_{1}}R\left[u\right]\;+\;{\vec{p}}^{\left(2\right)}\;\partial_{\xi_{2}}R\left[u\right]\right)=0.$$ (27)

Hence, *R*[*u*] is a (1+2)-dimensional structure. Its spatial dependence is confined to the plane defined by the space parts, ${\vec{p}}^{\left(1\right)}$ and ${\vec{p}}^{\left(2\right)}$, of the two 4-vectors.

The discussion in this paper focuses on pairs of fronts, for which *V*(*q*^(1)^, *q*^(2)^) > 0. Such pairs propagate rigidly at a velocities *v* < *c* = 1 (see Eq (16)). *R*[*u*] is then positive definite, bounded, has a maximum at *ξ*_1_ = *ξ*_2_ = −(log *V*(*q*^(1)^, *q*^(2)^))/2, and falls off exponentially as |*ξ*_*i*_| grow, e.g.:

$$R\left[u\right]\underset{\left|\xi_{2}\right|\to \infty }{\to }\frac{32\;e^{\xi_{1}}\;V\left(p^{\left(1\right)},\;p^{\left(2\right)}\right)}{\left(1\;+\;V\left(p^{\left(1\right)},\;p^{\left(2\right)}\right)\right)\;\left\{1\;+\;e^{2\;\xi_{1}}\;{\left(V\left(p^{\left(1\right)},\;p^{\left(2\right)}\right)\right)}^{2}\right\}}\;e^{-\left|\xi_{2}\right|}\;+\;O\left(e^{-2\;\left|\xi_{2}\right|}\right).$$ (28)

As a result, *R*[*u*] describes an infinitely long rod with a laterally bounded, positive definite profile. An example of the dependence of the profile, *R*[*u*], on the lateral coordinates is shown in Fig 5. (The case *V*(*p*^(1)^, *p*^(2)^) < 0 corresponds to pairs of fronts and to rods that propagate rigidly at velocities *v* > *c* = 1. *R*[*u*] is then also localized around the front intersection region. However, depending on the magnitude of *V*(*p*^(1)^, *p*^(2)^), its sign may vary, or it may be negative definite.)

Finally, a comment is due regarding the properties of a single-rod solution under Lorentz transformations, i.e., when *u* is a two-front solution of Eq (1). With a velocity, *v* < *c* = 1, *u* is a Lorentz scalar. Eq (2) implies that so is *R*[*u*]. Viewing *R*[*u*] as a mass density of the rod (by Eq (26), it is positive definite), the mass per unit length obeys the rules of relativity [10]:

$$\mu \equiv \int R\left[u\right]\;d^{2}{\vec{r}}_{⊥}=\frac{\mu_{0}}{\sqrt{1\;-\;v^{2}}}.$$ (29)

In Eq (29), *μ*_0_ is the rest-mass density per unit length, and ${\vec{r}}_{⊥}$ is the vector of spatial coordinates normal to the longitudinal axis of the rod. *v* is the velocity of propagation of the rod, which is perpendicular to the axis. *μ*_0_ is obtained through Eq (29) when the two-front solution is then at rest, *v* = 0. This happens when the two 4-mometa have vanishing time components:

$$p^{\left(i\right)}=\left(0,\;{\vec{n}}^{\left(i\right)}\right),\;{\vec{n}}^{\left(i\right)}\cdot {\vec{n}}^{\left(i\right)}=1,\;\left(i=1,\;2\right).$$ (30)

Technically, the computation of *μ*_0_ is simplest in a frame of reference, in which

$${\vec{n}}^{\left(i\right)}=\left(\text{cos}\varphi^{\left(i\right)},\text{ sin}\varphi^{\left(i\right)},\;0\right),$$ (31)

so that $d^{2}{\vec{r}}_{⊥}=dx\;dy$. The result is presented in Appendix B.

### 3.2 Multi-rod solutions

When the solution, *u*, of Eq (1) contains *N* ≥ 3 fronts, there are two possibilities.

If *all* $\left(\begin{array}{l} N\\ 3 \end{array}\right)$ 4-momentum triplets obey Eq (18), then *N*−2 of the vectors, *p*^(*i*)^, 1 ≤ *i* ≤ *N*, are linear combinations of two of them, say, *p*^(1)^ and *p*^(2)^. In particular, the space parts of *all N* 4-momenta lie in the plane defined by ${\vec{p}}^{\left(1\right)}$ and ${\vec{p}}^{\left(2\right)}$, the space parts of *p*^(1)^ and *p*^(2)^, and Eq (27) is obeyed. As a result, the whole solution moves rigidly in this plane; it is (1+2) dimensional. Its velocity of propagation is determined by Eq (16). Front intersections are then all perpendicular to the plane; all rods generated by *R*[*u*] are parallel. The number of distinct rods depends on the magnitudes of the free phase shifts in Eq (8), which determine the distances between rods. If all phase shifts vanish, then all fronts intersect along a single rod. When some phase shifts do not vanish, once the distances are appreciably greater than rod thicknesses, up to $\left(\begin{array}{l} N\\ 2 \end{array}\right)$ rods may become distinct. An example of a slower-than-light three-parallel-rod solution is shown in Fig 6.

If Eq (19) is obeyed by some 4-momentum triplet, then the pairwise intersection of the fronts yields three non-parallel rods. Eq (19) also guarantees that the longitudinal axes of the rods must intersect at a point. The condition for intersection is:

$$\xi_{1}\;+\;\delta_{1}=\xi_{2}\;+\;\delta_{2}=\xi_{3}\;+\;\delta_{3}=0,$$ (32)

where *ξ*_*i*_ are defined in Eq (8). Eq (19) guarantees that Eq (32) have a unique solution for the *t*-dependence of *x*, *y* and *z* of the intersection point:

$$x=\beta_{x}\;t\;+\;c_{x},\;y=\beta_{y}\;t\;+\;c_{y},\;z=\beta_{z}\;t\;+\;c_{z}.$$ (33)

Here *c*_*x*_, *c*_*y*_, and *c*_*z*_ are known constants and *β*_*x*_, *β*_*y*_ and *β*_*z*_ are defined in Eq (22). They obey Eq (25), so that the three-rod system propagates at the speed of light (*v* = 1). A three-rod solution of such type is shown in Fig 7.

In solutions of Eq (1) with *N* ≥ 4 fronts, the maximum number of rods is $\left(\begin{array}{l} N\\ 2 \end{array}\right)$. For example, consider the case of a four-front solution of Eq (1) (*N* = 4). If Eq (18) is obeyed by all four triplets of momentum vectors, then all the rods are parallel. Depending on the free phase shifts in Eq (8), some rods may coalesce, or not, the total number of parallel rods reaching at most six. If, on the other hand, Eq (19) is obeyed by one of the momentum triplets, then the corresponding triplet of rods are non-parallel and intersect at one point. The most complex structure that may then emerge is when the four intersections of triplets of the six rods make a tetrahedron in three-dimensional space. These intersection points move in space. At some finite time they become confined to a finite volume, which is determined by the free phase shifts in Eq (8). For example, they coalesce at *t* = 0 if all free phase shifts vanish. They move apart as *t* → ±∞.

## 4. Blobs

### 4.1 Construction and dynamical equation

In Section 3, it has been shown how to map spatially extended multi-front solutions onto infinitely long, laterally bounded, rods, which are localized in the vicinity of front intersections. In this Section the idea is extended to the rods themselves. A transformation of the general type of Eq (2) has not been found. However, a linear, parameter-dependent, operator, which maps multi-rod solutions onto structures that are localized around rod intersections, does exist. Its identification is based on the observation that, away from the longitudinal axis of a rod, its profile falls off exponentially fast (see Eq (28)).

**Fig 8:**
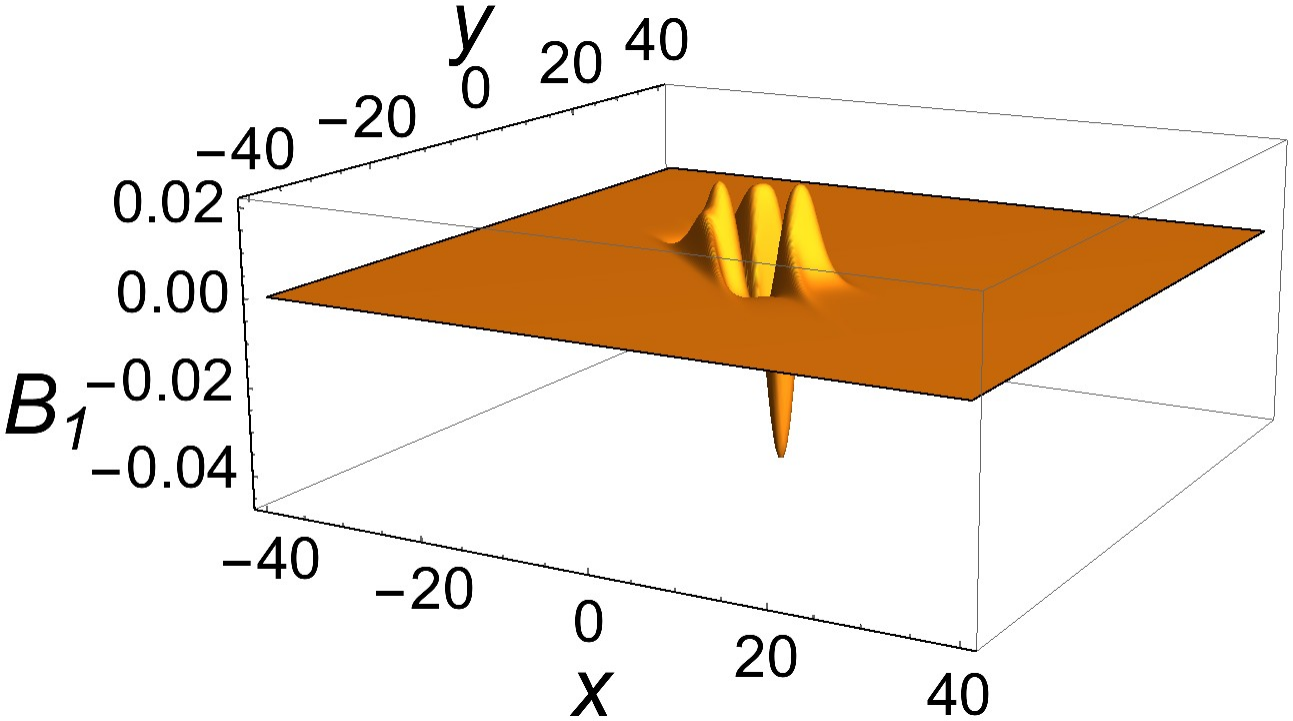
Profile of single-blob solution of Eq (35) at *t* = *z* = 0. Parameters as in Fig 7.

Consider a three-rod configuration, constructed out of momentum vectors, *p*^(1)^, *p*^(2)^ and *p*^(3)^, which obey Eq (19); the rods intersect around some point in space. The three-rod structure is a function the Lorentz scalars, *ξ*_1_, *ξ*_2_ and *ξ*_3_. Now, focus on one of the rods, say, the rod that is confined to the intersection region of the two fronts constructed from the vectors *p*^(1)^ and *p*^(2)^. Along that rod, far from the rod intersection region, the dependence on *ξ*_3_ falls off exponentially. In a similar manner, the dependence on *ξ*_1_ decays along the rod constructed out of *p*^(2)^ and *p*^(3)^, and the dependence on *ξ*_2_ disappears along the rod constructed out of *p*^(1)^ and *p*^(3)^. As a result, the entity,

$$B_{1}\left[u\right]=\partial_{\xi_{1}}\;\partial_{\xi_{2}}\;\partial_{\xi_{3}}R\left[u\right],$$ (34)

is confined to the intersection region of the three rods, and falls off exponentially in all directions in space away from that region. Owing to Eqs (33) and (25), such a blob propagates at the speed of light, *c* = 1. An example of a single blob is provided in Fig 8.

Finally, as the operation on *R*[*u*] in Eq (34) is linear, the blob obeys the linear wave equation, with the driving term of Eq (3) modified appropriately:

$$\partial_{\lambda }\partial^{\lambda }B_{1}\left[u\right]=\partial_{\xi_{1}}\;\partial_{\xi_{2}}\;\partial_{\xi_{3}}\left(\partial_{\mu }\partial_{\nu }u\;\partial^{\mu }\partial^{\nu }u\;-\;{\left(\partial_{\mu }\partial^{\mu }u\right)}^{2}\right).$$ (35)

The driving term on the r.h.s. of Eq (35) is localized in the vicinity of the rod intersection region. This can be demonstrated by direct computation.

Extension to solutions with *N* > 3 is straightforward. For each triplet of momentum vectors that generates three intersecting rods, one adds a derivative with respect to the corresponding triplet of Lorentz scalars. For example, in a four-front solution, there may be up to six front intersection regions. If all four triplets of 4-momentum vectors obey Eq (19), then *R*[*u*] of Eq (2) generates a six-rod structure, which is organized in triplets that intersect at four vertices. (The intersection points make a tetrahedron.) The resulting four-blob structure is generated by

$$B_{4}\left[u\right]=\left\{\partial_{\xi_{1}}\;\partial_{\xi_{2}}\;\partial_{\xi_{3}}\;+\;\partial_{\xi_{1}}\;\partial_{\xi_{2}}\;\partial_{\xi_{4}}\;+\;\partial_{\xi_{1}}\;\partial_{\xi_{3}}\;\partial_{\xi_{4}}\;+\;\partial_{\xi_{2}}\;\partial_{\xi_{3}}\;\partial_{\xi_{4}}\right\}R\left[u\right].$$ (36)

### 4.2 Elastic particle collisions?

When the blobs make a polyhedron in space (e.g., a tetrahedron in the case of *N* = 4 fronts), then each blob propagates at a velocity determined by Eqs (32) and (33). For *t* → −∞, the blobs are infinitely removed from one another. Around *t* ≅ 0, they become confined to some finite volume (for example, if all free phase shifts in Eq (8) are set to zero, the blobs all coalesce into one blob), and as *t* → +∞, they move apart and become infinitely removed from one another. This mimics an elastic collision amongst spatially extended particles.

### 4.3 |*t*| → ∞ and connection to unit sphere

All structures generated by Eqs (1), (3), (35) and (36) propagate at constant speeds. It, therefore, pays to study the solutions in the limit of |*t*| → ∞ in terms of scaled coordinates

$$\chi_{x}=\frac{x}{t},\;\chi_{y}=\frac{y}{t},\;\chi_{z}=\frac{z}{t}.$$ (37)

In the scaled coordinates, the fronts become sharp domain walls as |*t*| → ∞; each front has a width of *O*(1/*t*), which vanishes as |*t*| → ∞. The single-front solution obtained through Eqs (4)–(10), when expressed in terms of the scaled coordinates, provides a simple demonstration:

$$u=4\text{ arctan}\left[e^{t\;\left(p_{0}\;-\;p_{x}\;\chi_{x}\;-\;p_{y}\;\chi_{y}\;-\;p_{z}\;\chi_{z}\right)\;+\delta }\right].$$ (38)

Similarly, when studied in terms of the scaled coordinates, the rod solutions of Eq (3) shrink to lines of zero thickness, and the blobs representing rod intersection regions shrink to points of zero measure. Finally, using Eq (33), asymptotically in time, the scaled coordinates of a blob tend to:

$$\chi_{x}=\frac{x}{t}{|}_{\left|t\right|\to \infty }=\beta_{x},\;\chi_{y}=\frac{y}{t}{|}_{\left|t\right|\to \infty }=\beta_{y},\;\chi_{z}=\frac{z}{t}{|}_{\left|t\right|\to \infty }=\beta_{z}.$$ (39)

Thanks to Eq (25), the array of blobs (up to $\left(\begin{array}{l} N\\ 3 \end{array}\right)$ blobs in an *N*-front solution) freezes on the unit sphere as |*t*| → ∞. Triplets of parallel rods do not generate such points, they shrink to parallel lines that enter and exit the unit sphere.

## 5. Concluding comments

The goal of this paper has been to demonstrate through the example of the Sine-Gordon equation in (1+3) dimensions that it is possible to generate from a given nonlinear evolution equation, which has moving wave solutions with spatial extent, structures that have a greater level of spatial confinement, and obey an evolution equation of their own. This idea can be applied to many known evolution equations. The results will be reviewed in a separate publication.

## Appendix A: Properties of tachyonic momentum vectors under Lorentz transformations

The analysis addresses vectors that obey Eq (9). The goal is to show when such vectors can be Lorentz transformed into vectors with vanishing time components. Given a 4-momentum vector

$$p=\left(p_{0},\;p_{x},\;p_{y},p_{z}\right),$$ (A.1)

the Lorentz transformed vector is obtained by the transformation

$$p^{\prime }=L\;p,$$ (A.2)

where the matrix representation of *L* is written as:

$$L=\left(\begin{array}{cccc} \gamma & -\beta_{x}\;\gamma & -\beta_{y}\;\gamma & -\beta_{x}\;\gamma \\ -\beta_{x}\;\gamma & 1\;+\;\frac{\beta_{x}{}^{2}\;\left(\gamma \;-\;1\right)}{\beta^{2}} & \frac{\beta_{x}\;\beta_{y}\;\left(\gamma \;-\;1\right)}{\beta^{2}} & \frac{\beta_{x}\;\beta_{z}\;\left(\gamma \;-\;1\right)}{\beta^{2}}\\ -\beta_{y}\;\gamma & \frac{\beta_{x}\;\beta_{y}\;\left(\gamma \;-\;1\right)}{\beta^{2}} & 1\;+\;\frac{\beta_{y}{}^{2}\;\left(\gamma \;-\;1\right)}{\beta^{2}} & \frac{\beta_{y}\;\beta_{z}\;\left(\gamma \;-\;1\right)}{\beta^{2}}\\ -\beta_{x}\;\gamma & \frac{\beta_{x}\;\beta_{z}\;\left(\gamma \;-\;1\right)}{\beta^{2}} & \frac{\beta_{y}\;\beta_{z}\;\left(\gamma \;-\;1\right)}{\beta^{2}} & 1\;+\;\frac{\beta_{z}{}^{2}\;\left(\gamma \;-\;1\right)}{\beta^{2}} \end{array}\right),$$ (A.3)

$$\gamma =\frac{1}{\sqrt{1\;-\;\beta^{2}}},\;\beta =\sqrt{\beta_{x}{}^{2}\;+\;\beta_{y}{}^{2}\;+\;\beta_{z}{}^{2}}.$$ (A.4)

Single vector: One can always rotate the vector into one space dimension:

$$p=\left(p_{0},p_{x},0,0\right).$$ (A.5)

It, therefore, suffices to discuss a transformation in (1+1) dimensions, with *β*_*y*_ = *β*_*z*_ = 0. With

$$\beta_{x}=\frac{p_{0}}{p_{x}},$$ (A.6)

the transformed vector is

$$p^{\prime }=\left(0,\;1,\;0,\;0\right).$$ (A.7)

Thanks to Eq (9), one has |*β*_*x*_| < 1, so that the Lorentz transformation always exists.

Consider a single-front solution of Eq (1). *β*_*x*_ is the velocity of the front in the old frame of reference. In the transformed frame, the front solution of Eq (1) is at rest; owing to Eq (A.7), its profile is independent of the transformed time variable (see Eq (8)):

$$\xi =p_{\mu }x^{\mu }=p_{\mu }^{\prime }\cdot {x^{\prime }}^{\mu }=-{x^{\prime }}^{1}.$$ (A.8)

The same statement applies to any one front in a multi-front solution, once that front is far from front intersection regions, where it tends to a single-front solution.

Two vectors: One can always rotate the two vectors into two space dimensions

$$p^{\left(i\right)}=\left(p^{\left(i\right)}{}_{0},p^{\left(i\right)}{}_{x},p^{\left(i\right)}{}_{y},0\right),\;\left(i=1,\;2\right).$$ (A.9)

It, therefore, suffices to discuss a transformation in (1+2) dimensions, with *β*_*z*_ = 0. Applying the transformation of Eq (A.3) to the two vectors, one finds that the time components of both transformed vectors vanish for

$$\beta_{x}=\frac{p^{\left(1\right)}{}_{0}p^{\left(2\right)}{}_{y}\;-\;p^{\left(2\right)}{}_{0}p^{\left(1\right)}{}_{y}}{p^{\left(1\right)}{}_{x}p^{\left(2\right)}{}_{y}\;-\;p^{\left(2\right)}{}_{x}p^{\left(1\right)}{}_{y}},\;\beta_{y}=-\frac{p^{\left(1\right)}{}_{0}p^{\left(2\right)}{}_{x}\;-\;p^{\left(2\right)}{}_{0}p^{\left(1\right)}{}_{x}}{p^{\left(1\right)}{}_{x}p^{\left(2\right)}{}_{y}\;-\;p^{\left(2\right)}{}_{x}p^{\left(1\right)}{}_{y}}.$$ (A.10)

For *L* of Eq (A.3) to be a valid Lorentz transformation, one must have

$$\beta_{x}{}^{2}\;+\;\beta_{y}{}^{2}\leq 1.$$ (A.11)

This condition is obeyed when the scalar product of the two vectors in Minkowski space obeys

$$\left|p^{\left(1\right)}\cdot p^{\left(2\right)}\right|\leq 1.$$ (A.12)

The discussion following Eq (17) shows that the equality in Eq (A.12) is of no interest. Thus, when the magnitude of the scalar product is smaller than 1, a Lorentz transformation exists, which transforms both momentum vectors to ones that have vanishing time components. Consider now a two-front solution of Eq (1). By construction, it is (1+2) dimensional, propagating rigidly in the plane defined by ${\vec{p}}^{\left(1\right)}$ and ${\vec{p}}^{\left(2\right)}$, the space parts of the two vectors, at a velocity vector given by:

$$v=\left\{\beta_{x},\;\beta_{y}\right\}.$$ (A.13)

If a strict inequality is obeyed in Eq (A.12), then the two momentum vectors can be transformed to a frame of reference, in which they have vanishing time components, and by Eq (8), the two-front solution then does not depend on time. The two fronts are at rest. If Eq (16) is not obeyed, then the pair of fronts propagate rigidly at a velocity that exceeds *c* = 1.

Consider now a subset of 2 ≤ *m* ≤ *N* fronts in an *N*-front solution with *N* ≥ 3, when the subset is sufficiently far from intersections with all other fronts, so that it tends to an *m*-front solution. The statements made above apply to the subset if the momentum vectors corresponding to the *m* fronts all obey Eq (21), so that only two of the vectors are independent. The subset becomes (1+2)-dimensional. If Eq (A.12) is obeyed, it can be Lorentz transformed to a rest frame.

Three vectors: Given

$$p^{\left(i\right)}=\left(p^{\left(i\right)}{}_{0},p^{\left(i\right)}{}_{x},p^{\left(i\right)}{}_{y},p^{\left(i\right)}{}_{z}\right),\;\left(i=1,\;2,\;3\right),$$ (A.14)

With *β*_*x*_, *β*_*y*_ and *β*_*z*_ defined by Eqs (22) and (12), and obeying Eq (23), *L* of Eq (A.3) is a valid transformation of these three vectors into

$${p^{\prime }}^{\left(i\right)}=\left(0,{\vec{q}}^{\left(i\right)}\right),\;\left(i=1,\;2,\;3\right),$$ (A.15)

In general, it is not possible to transform more than three vectors into the form of Eq (A.15).

## Appendix B: Rest mass per unit length of rod

Using Eqs (29)–(31), *μ*_0_, the rest mass per unit length of a rod, is found to be given by:

$$\begin{array}{l} \mu_{0}=64\;\sqrt{V\left(p^{\left(1\right)},\;p^{\left(2\right)}\right)}\times \;\sum_{k=0}^{\infty }\frac{1}{{\left(2\;k\;+\;1\right)}^{2}}\;\frac{1}{{\left(1\;+\;V\left(p^{\left(1\right)},\;p^{\left(2\right)}\right)\right)}^{2\;k\;+\;1}}\;S\left(k,\;l\right)\\ S\left(k,\;l\right)=\sum_{l=0}^{k}\left(\begin{array}{c} 2\;k\;+\;1\\ 2\;l\;+\;1 \end{array}\right){\left(-1\right)}^{l}\;{\left(4\;V\left(p^{\left(1\right)},\;p^{\left(2\right)}\right)\right)}^{l}\;{\left(1\;-\;V\left(p^{\left(1\right)},\;p^{\left(2\right)}\right)\right)}^{2\;\left(k\;-\;l\right)}, \end{array}$$ (B.1)

where, using Eq (31), *V*(*p*^(1)^, *p*^(2)^) of Eq (10) is given by:

$$V\left(p^{\left(1\right)},\;p^{\left(2\right)}\right)={\left(\text{Tan}\left[\frac{\Delta \varphi }{2}\right]\right)}^{2},\;\left(\Delta \varphi =\left|\varphi^{\left(1\right)}\;-\;\varphi^{\left(2\right)}\right|\right).$$ (B.2)

*μ*_0_ is symmetric around **Δ***φ* = *π*/2, at which point it obtains the value

$$\mu_{0}{|}_{\Delta \varphi =\pi /2}=\mu_{0}=32\;\overset{\infty }{\underset{k=0}{\sum }}\frac{{\left(-1\right)}^{k}}{{\left(2\;k\;+\;1\right)}^{2}}=32\;G,$$ (B.3)

where *G* (≅ 0.915966) is the Catalan constant. The first few terms in the Taylor expansion of *μ*_0_ provide an excellent approximation for the entire range of 0 ≤ **Δ***φ* ≤ *π*/2:

$$\mu_{0}=16\;\Delta \varphi \text{ ln}\left[\frac{2\;e}{\Delta \varphi }\right]\;-\;\frac{4\;\Delta \varphi^{3}}{9}\;-\;\frac{7\;\Delta \varphi^{5}}{450}\;-\;\frac{31\;\Delta \varphi^{7}}{39690}.$$ (B.4)
